# Tuberculosis

**Published:** 1903-12

**Authors:** 


**Tuberculosis.** By Norman Bridge, M.D. Pp. 302. London: W. B. Saunders and Company. 1903.

In 295 pages Bridge has condensed a quantity of common-sense remarks on the question of Tuberculosis. He treats the disease as a whole, and does not spend much space on any of the indications of tuberculosis outside the chest, but he deals with phthisis as a type of the whole disease. This he divides into eight divisions, which are rather false distinctions from our point of view, and perhaps unnecessary; for although the types he describes are undoubtedly frequently to be met with, there are many cases which would not fall under any of these precise headings.

The writer is original in his teaching, and in more places than one differs from what is accepted by most. For instance, it is rather contrary to our received ideas to hear from him that catarrhs are rather likely to protect the tissues from contact with bacilli, and do no harm of any sort except to the convenience of the patient. Again, he holds that over-development of muscle or fat is always a misfortune, and is usually followed by a relapse of the tuberculosis, with reduced prospects of ultimate recovery. He pays a great deal of attention to indigestion, and on this account urges that the patient must be disabused of the notion that he is expected to eat a great deal at each meal: and he should be forbidden to take at any time a large meal: meals taken more frequently are better than large ones.

He is among those writers who accept apparently without question that the pulmonary apex can be infected through the lymphatic connections from the tonsils; and he urges against the sterilising of milk by boiling because of its "scorbutic" tendency.

He lays emphasis upon the relation of position to cough, which in his opinion may be diagnostic of phthisis. Thus when cough is excited by the patient's lying on the affected side it indicates that mucus is sinking back into smaller bronchioles, and this he believes to be almost indicative of phthisis. Lying on the other side, the mucus is expelled into larger bronchi, and produces no cough. This symptom ceases when the peripheral portion of lung is filled with disease products.

He deals more fully with prophylaxis and sanatorial treatment; in this he seems up-to-date, and yet he is not carried away out of the realms of common sense. This part of the book might with advantage be read by every practitioner and by many patients.

The style in places may be a little pithy and shows some want of arrangement of facts; but the book fulfils its purpose in a clear, readable way, and is presented to the reader in an acceptable form.

